# Socio-economic inequalities in health service utilization among Chinese rural migrant workers with New Cooperative Medical Scheme: a multilevel regression approach

**DOI:** 10.1186/s12889-022-13486-1

**Published:** 2022-06-03

**Authors:** Dan Li, Jian Zhang, Jinjuan Yang, Yongjian Xu, Ruoxi Lyu, Lichen Zhong, Xiao Wang

**Affiliations:** 1 School of Public Management, Northwest University, Xi’an, PR China; 2 School of Public Policy and Administration, Xi’an Jiaotong University, Xi’an, 710049 PR China; 3 School of Public Health, Health Science Center, Xi’an Jiaotong University, Xi’an, PR China; 4 International Business School Suzhou, Xi’an Jiaotong-Liverpool University, Xi’an, PR China

**Keywords:** Inequality, Health service utilization, Rural migrant workers, New Cooperative Medical Scheme, Multilevel regression approach

## Abstract

**Background:**

While reducing inequity in health service utilization is an important goal of China’s health system, it has been widely acknowledged that a huge number of rural migrant workers cannot be effectually protected against risks with the New Rural Cooperative Medical Insurance (NCMS).

**Method:**

Data of the 2016 China Labor-force Dynamic Survey and the Chinese Urban Statistical Yearbook were used. The multilevel regression approach was implemented with a nationally representative sample of rural migrant workers with NCMS. Our study adopted the concentration index and its decomposition method to quantify the inequality of their health service utilization.

**Result:**

The multilevel model analysis indicated that impact variables for health service utilization were not concentrated, especially the contextual and individual characteristics. The concentration indices of the probability of two weeks outpatient and the probability of inpatient were -0.168 (95%CI:-0.236,-0.092) and -0.072 (95%CI:-1.085,-0.060), respectively. The horizontal inequality indices for the probability of two-week outpatient and the probability of inpatient were -0.012 and 0.053, respectively.

**Conclusion:**

The health service utilization of rural migrant workers with NCMS is insufficient. Our study highlighted that substantial inequalities in their health service utilization did exist. In addition, their need of health service utilization increased the pro-poor inequality. Based on the findings, our study offered notable implications on compensation policies and benefit packages to improve the equality among rural migrant workers with NCMS.

## Background

Chinese rural migrant workers (also called “nongmingong”) have made a great contribution to the rapid urbanization and industrialization in China. However, the socially marginalized living condition in their urban residence caused by their dual identities, rural residents defined by the Chinese household registration system (hukou) working in urban areas, is hindering them to use public health services, which are more accessible for local urban residents. New Rural Cooperative Medical Scheme (NCMS), launched in 2003, has remarkably facilitated Chinese residents’ utilization of health services with a range of approaches, such as increasing the reimbursement ratio and upgrading the facility in primary medical institutions. However, rural migrant workers with NCMS still cannot be effectually protected against economic risks of diseases. The State Council called for the integration of the basic urban and rural medical insurance system in January 2016, but the newly launched Urban–Rural Resident Basic Medical Insurance (URBMI) has not been implemented thoroughly in China after its introduction. Therefore, it is meaningful to understand how to guide rural residents with URBMI or covered by the NCMS to seek medical treatment.

Previous studies have investigated the inequality in health service utilization, specifically focusing on NCMS. Che et al. [1] comparatively studied the inpatient situation in the NCMS pilot and non-pilot counties, and their findings showed that NCMS could eliminate the inequality of inpatient and inpatient expenses for rural residents but only to a limited extent. Han et al. [2] discovered that rural residents with lower income were more disadvantaged in using health services since the implementation of NCMS. Fang et al. [3] found that rural residents with higher income experienced a higher participation rate of NCMS, and NCMS promoted equality in health service utilization. Guo et al. [4] found that NCMS played a certain role in improving the incidence of compensation, but its effect was still limited in eliminating the economic burden of the rural residents. Li et al. [5] highlighted that the inequalities in the total cost and out-of-pocket cost of both outpatient and inpatient were evident among rural migrant workers with NCMS, and health service needs of the rural migrant workers with NCMS should be fully considered. With the goal of reducing inequality initiated by UN, the Chinese government focused on the healthcare service inequality by carrying out policies regarding basic public services. However, systematic research on the inequality in health service utilization of Chinese rural migrants with NCMS is far from sufficient.

There is abundant research on health service utilization and the impact factors of Chinese rural migrant workers’ health-seeking behaviors. For example, Peng et al. [6] studied the influence of socio-demographic characteristics on rural migrant workers’ decision to seek health care services when they fell ill and found that household monthly income per capita and daily working hours were directly proportional to their medical visiting rate. In addition, their results showed that health-seeking behaviors of rural migrants were significantly associated with their insurance coverage. Zhao et al. [7] found that the outpatient rate of middle-aged rural migrant workers in four weeks was 13.7% and its determinants included gender, marital status, income level, household size, the place of insurance enrollment, and self-assessed health (SAH). NCMS in China has obtained remarkable achievements through many difficulties and many rural migrant workers have been benefited. However, very little literature has explored whether the expected equality has been achieved and to what extent the inequality of health service utilization exists among rural migrant workers with NCMS.

This study involves three dimensions of Andersen model’s original version: predisposition, factors that enable or impede, and need for care. The Andersen model is a useful theoretical analysis framework with a wide range of variables to explain individual’s health service utilization [8–11]. The Andersen model (2013 Version) emphasizes the dynamics of and displays a conceptual model of health services use, namely, how contextual characteristics, individual characteristics, health behavior, and the health outcomes affect health service utilization. Some studies [12] have adopted the original Andersen model to explore the influencing factors on the health services utilization of rural migrants and have found that the current healthcare delivery system was not conducive for rural migrants to seek appropriate health services. However, few empirical studies in China have applied the Andersen Model (2013 Version) regarding its dynamic nature [13]. Most of related studies [6, 7] that have conducted descriptive or regression analysis could not fully display the unequal distribution of health service utilization among rural migrant workers with NCMS. In addition, the existing health services in China cannot satisfy the increasing needs of rural migrant workers, which were often neglected. The reason behind this mismatch was rarely explored. Further investigation on the contributors of inequality in health service utilization among rural migrant workers with NCMS is required. Hence, it is important that the needs of rural migrant workers with NCMS related to health service utilization are better grasped.

This study sought to explore the health service utilization of Chinese rural migrant workers by posing two major questions: 1) What are the factors that influence the health service utilization of rural migrant workers with NCMS? 2) Is there inequality in the health service utilization of rural migrant workers with NCMS? If the inequality exists, to what extent? Our findings can not only facilitate the mobility of rural migrant workers with NCMS, but also provide insights for improving health services to vulnerable groups.

## Methods

### Data

**Fig. 1:**
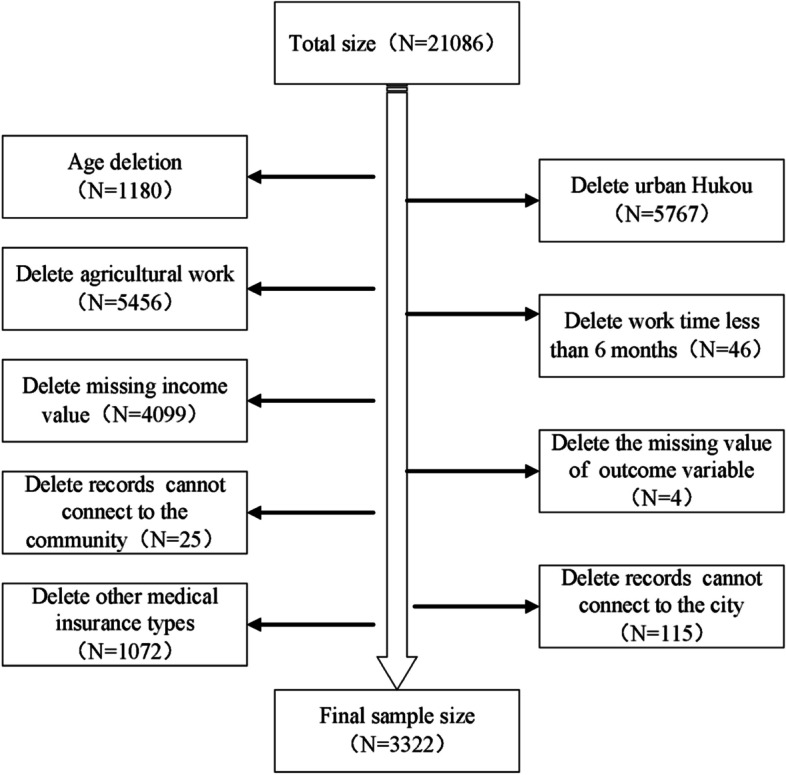
Screening process of sample in our study)

The data were derived from the 2016 China Labor-Force Dynamic Survey (CLDS 2016) published by the Center for Social Survey at Sun Yat-sen University and the data of the Urban Statistical Yearbook and Statistical Bulletin, covering detailed demographic, health, and economic situations, as well as health service utilization. The CLDS survey is a nationwide cross-sectional survey that targets China’s labor force. It adopts a multi-stage stratified sampling method, covering 29 provinces in China excluding Tibet and Hainan. The rotating-panel sample design adopted by the survey can well adapt to the drastic changes in Chinese society. The Data were collected from individuals, families in the remaining communities, and new communities in a new rotation group. While the data of CLDS 2016 were collected from 21,086 participants aged 15–64, our study focused on the rural migrant workers participating in NCMS in the same age group. Rural migrant workers, according to the commonly accepted definition, are the rural labor forces who engage in non-agricultural works and have worked outside their original (rural) areas for more than 6 months [14]. After data cleaning (i.e., excluding respondents with illogical answers or with key data missing), 3322 respondents were identified for further analysis (see Fig. 1). All analyses of the study were weighted using individual weights adjusted for non-response to obtain robust results.

### Measurement

Our study focused on the health service utilization of rural migrant workers participating in NCMS. Two questions in CLDS 2016 were used (originally in Chinese).

Question 1: Have you visited the clinic at least once in two weeks?

Question 2: Have you been admitted to the hospital during the past 12 months when you were sick or injured?

In this study, we adopted dummy variables with the value 1 if the respondent answered “yes”, and 0 if “no”.

### Predictors

To analyze the factors associated with health services utilization, we selected the predictors based on the Andersen Model (2013 Version). Our study only concerned how health services utilization is determined by four dynamics. In the Chinese socio-cultural context, we simplified the analysis framework considering the availability of data and the purposes of our study. We set parameters for the following variables of the conceptual framework:

1. Individual characteristics: age group (50 ~ 60, 61 and above), gender (male, female), living arrangement (living with spouse, living without spouse), educational attainment (below primary school, primary school, middle school and above), technical certificate (yes, no), type of industry (professional technician/clerical staff, service staff, manufacturing and construction, freelancer), type of employer (party/government institutions and state/collective-owned enterprises, private/foreign/joint venture, self-employed and freelancer), migration distance (within the county/district, cross the county/district), working hour (moderate labor, excessive labor [5]), income quintiles (poorest, poorer, middle, richer, richest), injury insurance (yes, no), number of friends (≤ 5, 6 ~ 10, ≥ 11), SAH (good, fair, poor).
2. health behavior: smoking (yes, no), alcohol use (yes, no), regular exercise per month (yes, no).
3. health outcome: the sense of fairness (unhappy, fair, happy).
4. contextual characteristics: the proportion of ethnic minorities (per capaita in the community) service quality index of the community, region (east, central, west), city level which reflecting the political rule, socio-economic development and the policy-oriented factors in China (below sub-provincial city, sub-provincial city and above), service quality index of the city, health index of the community, the number of medical institutions per 10,000 people in the community, the number of medical institutions per 10,000 people in the city, the number of hospital beds per 10,000 people in the city, and the number of doctors per 10,000 people in the city.

### Multilevel regression approach

We used the nationally representative date in this study, which shown a obvious hierarchical structure. To capture within-group and between-group correlations in calculation, we estimated a series of three-level regression approaches, in which rural migrant workers with NCMS were nested within communities and cities because the data showed a hierarchical structure of “city-community-rural migrant workers with NCMS”. As noticed by Neuhaus et al. [15] and Snijders et al. [16], in a multilevel context, the relationships at the cluster level, measured by the between-cluster effects, can be very different from the relationships at the micro-level, measured by the within-cluster effects. For instance, rural migrant workers with NCMS in the same city or community may have the same city characteristics or community characteristics. Furthermore, due to similar living environment, the differences between rural migrant workers with NCMS living in the same community is less than those living in different communities. Those violates the classical assumption of the independence of error term in a single level regression model and the “mean square deviation” of city-level or community-level. When data are sampled in multi-level, failing to consider the clustering of the observations and ignoring the hierarchical structure of the data can lead to false inferences being drawn from the data.

Intra-class Correlation Coefficient (ICC) is the ratio of the between-group variance to the total variance, representing the degree of variation between groups. The calculation formula of ICC is as follows:

1 $$\mathrm{ICC}=\frac{{\sigma }_{u0}^{2}}{{\sigma }_{u0}^{2}+{\sigma }_{e0}^{2}}$$

${\sigma}_{u0}^{2}$ presents the between-group variance and ${\sigma }_{e0}^{2}$ presents the within-group variance. When ICC is closer to 0, the rural migrant workers with NCMS in the group tend to be independent, which represents that the multilevel model can be simplified to a fixed-effect model; when the ICC is closer to 1, the difference between groups is larger than that within the group. When ICC is significantly larger than 0.059, multilevel regression models should be considered [17]. In addition, decreases in variance and model fit statistics (for example, AIC and BIC) indicate a good performance[18]. When the dependent variable is a binary variable, a linear approximation method in the generalized linear model needs to be used.

On the model establishment, the basic operation steps of multilevel models are as listed below: First, establish a null model, which is also known as an unconditional two-level model, to check the hierarchical structure of the data. ICC can be utilized to judge whether it can be used for analysis the multi-level data. Secondly, include variables representing the fixed effects to expand the null model to observe the significance of high-level explanatory variables. Thirdly, include the explanatory variable in level 1. The random slope of level 1 can be tested to adjust the effect of the level of rural migrant workers with NCMS.

The three-level logistic regression model is expressed as follows:

2 $$\mathrm{logit}\left(\frac{{P}_{ijk}}{1-{P}_{ijk}}\right)={\beta x}_{ijk}+{\gamma w}_{jk}+{\tau z}_{k}+{\mu }_{jk}+{v}_{k}$$

where *i*, *j*, and *k* represent level 3-city, level 2-community, and level 1-rural migrant workers with NCMS. ${x}_{ijk}$, ${w}_{jk}$ and ${z}_{k}$ represent the explanatory variables of level 1-rural migrant workers with NCMS, level 2-community, and level 3-city, respectively. $\beta$, $\upgamma$, and $\tau$ represent the estimated value of the regression coefficient of the explanatory variable at each level. ${\mu }_{jk}$ and ${v}_{k}$ represent the residuals of level 2-community and level 3-city, respectively.

The three-level regression model is expressed as follows:

3 $${y}_{ijk}={\beta x}_{ijk}+{\gamma w}_{jk}+{\tau z}_{k}+{\mu }_{jk}+{v}_{k}$$

${y}_{ijk}$ is a continuous dependent variable. *i*, *j*, and *k* represent level 3-city, level 2-community, and level 1-rural migrant workers with NCMS.${x}_{ijk}$,${w}_{jk}$, and ${z}_{k}$ represent the explanatory variables of level 1-rural migrant workers with NCMS, level 2-community, and level 3-city, respectively. $\beta$,$\upgamma$, and $\tau$ represent the estimated value of the regression coefficient of the explanatory variable at each level. ${\mu }_{jk}$ and ${v}_{k}$ represent the residuals of level 2-community and level 3-city, respectively. The three-level regression model in our study addressed the first question.

### Concentration index and decomposition

The inequality of health service utilization across socio-economic groups was estimated using a concentration index (CI). The CI is defined as twice the area between the concentration curve and the line of equality. When it takes values between -1 and 1, where a positive value indicates that a variable is more concentrated among richer rural migrant workers with NCMS and a negative value indicates less [19, 20]. The formula for computing the CI is:

4 $$\mathrm{CI}=\frac{2}{\mu } \mathrm{cov }\left({y}_{i},{R}_{i}\right)$$

where CI is the concentration index of health service utilization of rural migrant workers with NCMS,${y}_{i}$ is the health service utilization indicators, μ is the mean of health service utilization, and ${R}_{i}$ is the fractional rank in the economic status distribution. The inequalities in two-week outpatient probability and inpatient probability among rural migrant workers with NCMS were measured by CIs. The CIs helped us to measure the degree of inequality in health service utilization of rural migrant workers with NCMS, which addressed the second question.

Decomposition methods can quantify each determinant’s specific contribution to the measured income-related inequality while controlling for other determinants, providing a basis for prioritizing interventions [21, 22]. The decomposition shows how each determinant’s separate contribution to explained income-related inequality can be decomposed into its elasticity and its income-related inequality. That is, each contribution is the product of the sensitivity of health service utilization with respect to that factor and the degree of income-related inequality in that factor. The decomposition of the CI clarified the need of health service utilization, to prepare for a further answer to the third question. As the probability of health service utilization is a dummy variable, a generalized linear model with binomial distribution and identity link was employed. The regression model is as follows:

5 $$\mathrm{y}={\alpha }^{m}+{\sum }_{j}{\beta }_{j}^{m}{x}_{j}+\varepsilon$$

where y is the health service utilization indicator, ${\beta }_{j}^{m}$ is the partial effects (i.e., dy/dxj) of each variable and evaluated at sample means,${\alpha }^{m}$ is the constant term in the regression equation, $\upvarepsilon$ is the error term. Calculating the CI of Eq. (2) and the decomposition of the CI could be specified as:

6 $$\mathrm{CI}={\sum }_{j}({{\beta }_{j}^{m}}\left/ {\mu}\right.){C}_{j}+G{C}_{\varepsilon }$$

where $\upmu$ is the mean of the dependent variable, ${C}_{j}$ is the concentration index for ${x}_{j}$,${{\beta }_{j}^{m}}\!\left/ \!{\mu }\right.$ is the elasticity of ${x}_{j}$ in health service utilization of rural migrant workers with NCMS, and G is the elasticity of $\upvarepsilon$ in health service utilization. The contribution of ${x}_{j}$ is defined as the product of the elasticity of ${x}_{j}$ in health service utilization and the CI of ${x}_{j}$. The large elasticity of health service utilization with respect to these factors is responsible for their large contribution to the CI of health service utilization. The positive contribution of one factor indicated the factor widened the pro-rich (pro-poor) inequality, and vice versa.

To clarify the need for health service utilization, the horizontal inequality index (HI) was calculated considering the need for health service utilization among rural migrant workers with NCMS. In this study, HI of health service utilization was measured by deducting the contributions of unavoidable variables (such as gender, age, and SAH) from the overall CI. A positive (negative) HI also indicated the pro-rich (pro-poor) inequality. The results of HI are also conducive to the second question. The formula is as follows:

7 $$\mathrm{HI}=\mathrm{CI}-{\textstyle\sum_j}(\beta_j^mx_{ji}/\mu)C_j$$

${\beta }_{j}^{m}$ presents the partial regression coefficient of the variable of health service needs.${x}_{j}$ and ${C}_{j}$ present the mean and the CI of health service need.$\mu$ presents the mean of y. The need variables of health service utilization in our study were age, gender and SAH.

All analyses were performed with STATA 15.0 (StataCorp LP., College Station, TX, USA). The probability, a *p*-value of less than 0.05 was considered statistically significant. We used the “mean replacement method” to deal with missing data as less than 15% of the data were missing for each variable in our analysis.

## Results

Table 1 presents the variables and the descriptive statistics. within rural migrant workers with NCMS. Among the 3322 respondents, 210 (6.32%) and 196 (5.90%) experienced two-week visits to clinics and hospitals during the past 12 months respectively.

**Table 1:** Statistics for the characteristics of respondents

Variables	Number/Mean	Percentage/SD
**Outcome Variables**		
Two-week outpatient		
Yes^†^	210	6.32
No	3112	93.68
Inpatient probability		
Yes^†^	196	5.90
No	3126	94.10
**Individual characteristics**		
Age group		
15 ~ 36^†^	1303	39.22
36 ~ 50	1199	36.09
50 ~ 64	820	24.68
Gender		
Men^†^	1910	57.50
Women	1412	42.50
Living arrangement		
Live with spouse^†^	500	15.05
Live without spouse	2822	84.95
Educational attainment		
Below primary school^†^	923	27.78
Primary school	1619	48.74
Middle school and above	780	23.48
Technical certificate		
Yes^†^	422	12.70
No	2900	87.30
Type of industry		
Professional technician/Clerical staff^†^	248	7.47
Service stuff	1177	35.43
Manufacturing and construction	1041	31.34
Freelancer	856	25.77
Type of unit		
Party/government/state-owned^†^	300	9.03
Collective enterprises and institutions	1327	39.95
Self-employed and freelance	1695	51.02
Working hours		
Moderate labor^†^	1471	44.28
Excessive labor	1851	55.72
Place of work		
In the county/district^†^	2721	81.91
Across the county/district	601	18.09
Income quintiles	664	19.99
Poorest^†^	665	20.02
Poorer	664	19.99
Middle	665	20.02
Richer	664	19.99
Richest	664	19.99
Injury insurance		
Yes^†^		
No	293	8.82
number of friends	3029	91.18
< = 5^†^		
6 ~ 10	1904	57.31
> = 11	811	24.41
SAH	607	18.27
Good^†^		
Fair	2285	68.78
Poor	837	25.20
**health behavior**		
Smoke		
Yes^†^	1192	35.88
No	2130	64.12
Alcohol use		
Yes^†^	831	25.02
No	2491	74.98
Regular exercise every month		
Yes^†^	818	24.62
No	2504	75.38
**Health outcome**		
Sense of happiness		
Unhappy^†^		
Fair	215	6.47
Happy	1014	30.52
**Contextual characteristic**		
Proportion of ethnic minorities	1.000	0.006
Per capita in the community	1.000	2.02 × 10^–4^
Region		
East^†^	2074	62.43
Middle	639	19.24
West	609	18.33
City level		
Sub-provincial city and above	570	17.16
Other	2752	82.84
Number of medical institutions for 10,000 people in the community	5.60	18.48
Number of medical institutions for 10,000 people in the city	2601.65	4597.18
Number of doctors for 10,000 people in the city	7.48	12.24
Number of beds for 10,000 people in the city	0.70	1.33
Health index of the community	54.34	19.24
Service quality index of the community	83.94	44.33
Urban service quality index	-0.05	0.64
Intercept	0.07	0.24

*SD* standard deviation

^†^Reference levels in the regressions

Table 2 presents that the community-level variance of the two-week outpatient probability and inpatient probability is 0.350 and 0.065 respectively. The community-level ICCs were calculated to be 0.096 and 0.019 respectively. The model fit statistics of the two-week outpatient probability (AIC = 1545.489, BIC = 1557.703) and the inpatient probability (AIC = 1487.371, BIC = 1499.585) were examined.

**Table 2:** Two empty model of influencing factors of health service utilization

Variables		Two-week outpatient service		Inpatient service	
		OR	SE	OR	SE
Fixed effects					
	Intercept	-2.877***	0.105	-2.796***	0.092
Random effects					
	Community level variance	0.350	0.141	0.065	0.124
	Personal level parameter	1.000	0.000	1.000	0.000

Estimates of random-effect parameters and residual variance parameters were reported as standard errors. OR for odds ratio; SE for standard error; *p < 0.05, **p < 0.01, ***p < 0.001

Table 3 presents the estimations of the three-level regression models. The city- and community-level variances of the two-week outpatient probability are 0.243 and 0.036 respectively. Then, city- and community-level ICCs were calculated to be 0.056 and 0.085 respectively. The model fit statistics of the two-week outpatient probability were examined for the models (AIC of 1362.727 and BIC of 1624.35). Both variances and model fit statistics decreased, which indicating good performance. Therefore, a multilevel regression model was appropriate to analyze the probability of two-week outpatient among rural migrant workers with NCMS. The city- and community-level variances of the inpatient probability were found to be 4.07 × 10^–33^ and 0.067, respectively. Then, the city- and community-level ICCs are calculated to be 1.21 × 10^–33^ and 0.019, respectively. The model fit statistics of the inpatient probability were examined for the models (AIC = 1383.709, BIC = 1639.247). As ICC of the inpatient probability at the city and community level is significantly smaller than 0.059 [17], it is unnecessary to use a multilevel regression model to analyze the probability of inpatient among rural migrant workers with NCMS.

**Table 3:** Three-level empty model of influencing factors of health service utilization

Variables		Two-week outpatient service		Inpatient service	
		OR	SE	OR	SE
Fixed effects					
	Intercept	-2.873***	0.112	-2.815***	0.094
Random effects					
	City level variance	0.243	0.129	4.07 × 10^–33^	2.18 × 10^–17^
	Community level variance	0.036	0.147	0.067	0.129
	Personal level parameter	1.000	0.000	1.000	0.000

Estimates of random-effect parameters and residual variance parameters were reported as standard errors. OR for odds ratio; SE for standard error; *p < 0.05, **p < 0.01, ***p < 0.001

As Table 4 shows, the rural migrant workers living with a spouse were more likely to visit clinics at least once within two weeks than those without a spouse(OR = 0.539, *P* < 0.01). The two-week outpatient probability of rural migrant workers with fair SAH and poor SAH was found to be significantly higher than for those with good SAH (OR = 3.947, *P* < 0.01; OR = 14.608, *P* < 0.01). The inpatient probability was significantly higher for female rural migrant workers than for those who were male (OR = 1.753, *P* < 0.05). The inpatient probability of rural migrant workers with NCMS in the county/district was higher than for those across the county/district (OR = 1.689, *P* < 0.01). Regarding SAH, the inpatient probabilities of rural migrant workers with fair SAH and poor SAH was significantly higher than for those with good SAH (OR = 2.462, *P* < 0.001; OR = 8.280, *P* < 0.001). The inpatient probability of rural migrant workers who did not participate in regular exercise was significantly lower than for those with regular exercise who did (OR = 0.690, *P* < 0.05).

**Table 4:** Association of independent variables and health service utilization

Variables	Two-week outpatient probability		Inpatient probability	
	OR	SE	OR	SE
**Individual characteristics**				
Age group				
15 ~ 36^†^				
36 ~ 50	1.010	0.219	0.724	0.152
50 ~ 64	1.001	0.247	1.197	0.263
Gender				
Men^†^				
Women	0.865	0.182	1.753*	0.398
Living arrangement				
Live with spouse^†^				
Live without spouse	0.539**	0.119	1.231	0.310
Educational attainment				
Below primary school^†^				
Primary school	1.363	0.269	1.277	0.242
Middle school and above	1.087	0.291	1.140	0.287
Technical certificate				
Yes^†^				
No	0.967	0.261	0.679	0.164
Type of industry				
Professional technician/Clerical staff ^†^				
Service stuff	0.862	0.305	1.404	0.553
Manufacturing and construction	0.720	0.267	1.721	0.698
Freelancer	0.532	0.219	1.378	0.606
Type of unit				
Party/government/state-owned^†^				
Collective enterprises and institutions	1.107	0.369	1.273	0.430
Self-employed and freelance	1.318	0.459	1.234	0.434
Working hours				
Moderate labor^†^				
Excessive labor	1.091	0.180	0.920	0.145
Place of work				
In the county/district^†^				
Across the county/district	0.867	0.127	1.689**	0.416
Income quintiles				
Poorest^†^				
Poorer	0.884	0.230	1.037	0.239
Middle	0.695	0.186	0.761	0.191
Richer	0.824	0.316	1.025	0.256
Richest	0.612	0.182	1.151	0.303
Injury insurance				
Yes^†^				
No	0.616	0.169	1.289	0.405
number of friends				
< = 5^†^				
6 ~ 10	1.061	0.220	0.790	0.156
> = 11	0.506	0.170	1.054	0.220
SAH				
Good^†^				
Fair	3.947***	0.889	2.462***	0.442
Poor	14.608***	3.182	8.280***	1.872
**health behavior**				
Smoke				
Yes^†^				
No	1.429	0.312	0.332	0.331
Alcohol use				
Yes^†^				
No	1.168	0.264	0.363	0.292
Regular exercise every month				
Yes^†^				
No	0.759	0.137	0.109	0.252
**Health outcome**				
Sense of happiness				
Unhappy^†^				
Fair	0.894	0.236	0.956	0.293
Happy	0.697	0.184	1.267	0.372
**Contextual characteristic**				
Proportion of ethnic minorities	1.005	0.004	1.006	0.003
Per capita in the community	1.000	1.10 × 10^–5^	0.929	9.65 × 10^–5^
Region				
East^†^				
Middle	1.351	0.402	0.876	0.204
West	1.062	0.345	0.934	0.246
City level				
Sub-provincial city and above				
Other	1.345	0.390	1.166	0.260
Number of medical institutions for 10,000 people in the community	2.32 × 10^–30^	2.17 × 10^–28^	0.994	0.008
Number of medical institutions for 10,000 people in the city	1.011	0.097	1.025	0.084
Number of doctors for 10,000 people in the city	1.000	0.003	0.989	0.002
Number of beds for 10,000 people in the city	0.991	0.006	0.995	0.005
Health index of the community	0.892	0.145	0.994	0.128
Service quality index of the community	1.089	0.129	1.078	0.113
Urban service quality index	0.973	0.209	0.963	0.178
Intercept	0.108	0.089	0.015**	0.013

The Symbol of “*” is defined by a *p* value < 0.05; the Symbol of “**” is defined by a *p* value < 0.01; the Symbol of “***” is defined by a *p* value < 0.001

^†^Reference levels in the regressions

Table 5 shows the two-week outpatient probability and inpatient probability among rural migrant workers with NCMS in different economic quintiles in China. The two-week outpatient probabilities among rural migrant workers with NCMS in the five economic groups was 9.74%, 6.61%, 6.25%, 6.61%, and 3.13%, respectively. While, the proportions of inpatient probability in these five economic groups was found to be7.30%, 7.48%, 4.86%, 5.39%, and 6.26%, respectively.

**Table 5:** Health service probability in different economic quintiles in China

Economic Quintiles	Two-week outpatient probability		Inpatient probability	
	Number	Percentage (%)	Number	Percentage (%)
Poorest	56	9.74	42	7.30
Poorer	38	6.61	43	7.48
Middle	36	6.25	28	4.86
Richer	38	6.61	31	5.39
Richest	18	3.13	36	6.26
All	210		196	

The CI of two-week outpatient probability was -0.020 (95% confidence interval: -0.236, -0.092) and the CI of inpatient probability was -0.072 (95% confidence interval: -1.085, -0.060). The significantly negative values of CIs indicated strong pro-poor inequalities, that is, the richer rural migrant workers with NCMS used fewer health service than the poor. The inequalities in health service utilization can be further explained by decomposing the CIs into the determined components. dy/dx and the percentage of contribution (%) of each determined component are presented in Table 6. The factors found that contributed to the inequality in two-week outpatient probability were as follows: female (85.41%), living without a spouse (27.17%), richer group (-36.47%), poor SAH (54.76%), the number of hospital beds per 10,000 people (44.98%), and service quality index of the city (-35.79%). It meant that female, living without a spouse, poor SAH and the number of hospital beds per 10,000 people increased the pro-poor inequality in two-week outpatient probability, but richer group and service quality index of the city reduced the pro-poor inequality in two-week outpatient probability. The factors that contributed most to the inequality of inpatient probability were female (53.85%), richest group (-45.53%), fair SAH (22.03%), and poor SAH (89.01%). It means that female, fair SAH and poor SAH increased the pro-poor inequality in inpatient probability, but the richest group reduced the pro-poor inequality in inpatient probability.

**Table 6:** Decomposition of concentration index of two-week outpatient probability and inpatient probability among rural migrant workers with NCMS

	Two-week outpatient probability		Inpatient probability	
	dy/dx	Contributions/%	dy/dx	Contributions/%
36 ~ 50	0.001	-0.26	-0.076	5.22
50 ~ 64	0.069	5.82	0.030	3.20
Women	0.167	85.41	0.217	53.85
Live without spouse	-0.315	27.17	0.137	-2.84
Primary school	0.270	-13.98	0.100	-5.44
Middle school and above	-0.019	3.44	0.014	-1.23
Having technical certificate	0.161	-2.57	-0.432	-13.31
Service stuff	0.296	-10.41	0.133	-9.63
Manufacturing and construction	0.382	6.01	0.143	1.49
Freelancer	0.271	2.34	0.121	10.05
Collective enterprises and institutions	-0.234	-7.79	0.074	0.59
Self-employed and freelance	-0.488	0.54	-0.003	0.07
Excessive labor	0.140	-28.00	-0.017	1.06
Across the county/district	-0.225	8.91	0.233	9.65
Poorer	0.042	9.16	-0.027	-11.57
Middle	-0.026	7.32	-0.016	2.53
Richer	0.077	-36.47	-0.014	9.73
Richest	0.036	-10.78	0.040	-45.53
Having Injury insurance	-0.968	-9.12	-0.051	-1.60
Number of friends 6 ~ 10	-0.093	13.22	-0.051	2.51
Number of friends > = 11	0.127	-10.82	0.004	-0.54
Fair SAH	-0.086	6.93	0.233	22.03
Poor SAH	0.110	54.76	0.226	89.01
No Smoking	-0.118	-14.83	-0.106	-13.50
No alcohol use	0.044	6.62	-0.096	-8.47
No regular exercise every month	-0.389	-5.87	-0.209	-6.07
Fair happiness	0.149	5.43	-0.031	-0.95
Happy	0.313	-14.15	0.085	-3.17
Proportion of ethnic minorities	-0.005	-0.39	0.030	5.67
Per capita in the community	0.024	-9.43	0.011	-2.29
Middle	0.093	-8.02	0.061	-4.27
West	-0.015	-7.75	-0.019	-1.20
Below Sub-provincial city	0.614	5.62	0.166	0.73
Number of medical institutions for 10,000 people in the community	-0.086	11.04	0.009	0.50
Number of medical institutions for 10,000 people in the city	0.152	-2.85	-0.025	1.43
Number of beds for 10,000 people in the city in the city	-0.677	44.98	0.015	-0.57
Health index of the community	-0.010	-7.69	0.000	-1.07
Service quality index of the community	-0.008	-13.20	0.004	-1.48
Service quality index of the city	-0.024	-35.79	0.006	4.17

As Table 7 shows, the contribution rates of need to the inequalities of two-week outpatient probability and inpatient probability were 40.80% and 173.30% respectively. The HIs of the two-week outpatient probability inequality and the inpatient probability inequality were -0.012 and 0.053, respectively. The HIs were higher than the CIs indicating that accounting for need for health services reduces the magnitude of the pro-poor inequality. In the case of inpatient probability, the index moved from negative to positive indicating a change to a slight pro-rich inequality.

**Table 7:** Horizontal index of two-week outpatient probability and inpatient probability among rural migrant workers with NCMS

	Two-week outpatient probability			Inpatient probability	
	CI	Contributions/%		CI	Contributions/%
CI	-0.020	100.00		-0.072	100.00
Need	-0.008	40.80		-0.125	173.30
Economy	-0.003	14.57		0.032	-44.84
Other	-0.007	31.87		0.031	-42.95
residual	-0.003	12.76		-0.010	14.49
HI	-0.012			0.053	

*CI* for concentration index, *HI* for horizontal inequality index

## Discussion

Reducing inequalities has been widely recognized as a major objective of health policies in China, and has become a growing concern of the public. As major medical insurance for rural migrant workers, NCMS has improved their medical treatment. Although China has the largest scale of population migration, there are few studies on the inequality in health service utilization among rural migrant workers with NCMS. To gain a better understanding of health service utilization regarding rural migrant workers with NCMS, we selected and used predictors from the Andersen Model (2013 Version) in the Chinese socio-cultural context to enhance its explanatory power when applied to empirical studies. Our results were conducive to an objective and comprehensive understanding of the health service utilization of rural migrant workers with NCMS.

The Fifth Chinese National Health Service Survey [23] showed that the two-week outpatient and inpatient probabilities of Chinese residents were 8.17% and 7.78% respectively. The China Health and Retirement Longitudinal Study [6] showed that the four-week outpatient probability was 13.7%, and the average cost was 400.3 yuan. The Chinese government made a comprehensive deployment of the milestone strategy of “Healthy China” to prioritize people’s health by integrating health into all policies [24]. Our results shown that the two-week outpatient probability (6.32%) and inpatient probability (5.90%) for rural migrant workers with NCMS were much lower than those for the general population as determined in CLDS 2016 (6.38% and 7.52% respectively). It can be seen that the health service utilization of rural migrant workers with NCMS was lower than that of the overall Chinese labor force. The Chinese government should spare more coordinated and comprehensive efforts to ensure people’s equal access to health services, especially that of rural migrant workers. In line with previous studies [5, 25], the current health service system discouraged rural migrant workers from seeking appropriate care of good quality. Combined with this factor and others, such as: the lack of specific implementation rules for NCMS, low income, frequent job changes, and high work intensity, have led to poor health service utilization by those workers.

In addition to offering a reasonable and reliable analysis framework for explaining health service utilization and its inequality among rural migrant workers with NCMS in China, this study also has significance in terms of application the Andersen Model (2013 Version) in the field of health in China. Our results revealed a variety of variables associated with the two-week outpatient probability and inpatient probability among rural migrant workers with NCMS in China. Therefore, we should pay attention to the health education of rural migrant workers and guide them to take regular physical exercise. It was found that rural migrant workers with stable jobs and income tended to use more health services. In addition, employers should approve sick leaves for those who need medical treatment to receive timely treatment. Our study, as well as previous studies [26], revealed that the inpatient probability is unequally distributed among the income spectrum. Our analyses provide evidence for the existence of pro-poor inequality that the poor are more likely to utilize health services. According to previous studies [27], rural migrant workers with higher economic status had better SAH, resulting in less need for hospitalization.

Adopting the theory of equal opportunity, we fully considered the need of rural migrant workers with NCMS for health services. The inequality in two-week inpatient probability, compared with the inequality in two-week outpatient probability, was found to be lower. This is related to the hospitalized compensation plan in China. Rural migrant workers are generally at a disadvantage in the labor market, and some of them are engaged in physical labor with high work intensity. Although the quality of health services for rural migrant workers improved, the predicaments faced by those workers have not been eliminated and their demand for health services has not been met. Since the 19th CPC National Congress in 2016, the Chinese government has been given increasing focus to the health needs of rural migrant workers, but joint efforts by society are still needed to improve their health in the long run.

The results highlight that gender, marital status, economic level, SAH, number of hospital beds per 10,000 urban population, and urban service quality index are the main contributing factors in relation to the inequality in two-week outpatient probability. The family support provided [28] helped improve the health status of rural migrant workers, thus reducing the probability of their seeking medical treatment. Poor SAH increased inequality in favor of a higher two-week outpatient probability for rural migrant workers with a lower economic status. Most rural migrant workers obtain a higher income by engaging in intensive physical work, so healthier rural migrant workers with NCMS are more likely to obtain a higher income. Gender, economic level, and SAH are the key indicators for the inequalities in the inpatient probability. The childbirth needs of female rural migrant workers for childbearing age exacerbate inequality. General SAH increases the inequality of inpatient probability of migrant workers with a lower income. Those findings are consistent with the existing research [29]. The proportion of inpatient reimbursement was higher compared to outpatient reimbursement. The urban service quality index and the number of hospital beds per 10,000 in the cities reflect that cities attach great importance to the livelihood of people, which can promote the level of medical insurance and thus help to improve the medical treatment of rural migrant workers. This results in an increasing recognition that reducing inequality in healthcare service utilization is a critical issue to address.

This study also have several limitations. First, due to the cross-sectional analysis, the determination of time precedence or causal inferences cannot be solved. More studies are needed to further explore a causal inference for specific key factors. Second, considering the availability of data, our study does not allow complete testing of the Chinese construction for the Andersen model. Third, the limited sample size for the rural migrant workers could not represent the most current statistics and may lead to underestimated or overestimated regarding results.

## Conclusion

In conclusion, our study sheds light on the inequalities in the health service utilization of rural migrant workers with NCMS in China. Our findings provide evidence for the pro-poor inequality in regard to two-week outpatient and inpatient probabilities. These findings illustrate the main determinants of inequality in health service utilization and highlight the important influencing factors–- gender, marital status, economic status, SAH, number of beds per 10,000 population, and the urban service quality index. Our study found that if we do not take the health service needs into account, we may overestimate or underestimate the inequality in the health service utilization of rural migrant workers with NCMS. Thus, it is essential to involve rural migrant workers’ needs for offering better-designed health services to rural migrant workers.

## Data Availability

The data used in our study can be applied and obtained from the 2016 CLDS of Center for Social Survey, Sun Yat-sen University (available online:http://css.sysu.edu.cn/Data).

## Abbreviations

Hukou: Chinese household registration system
NCMS: New Rural Cooperative Medical Insurance
URBMI: Urban–rural Resident Basic Medical Insurance
CLDS 2016: China Labor-Force Dynamic Survey in 2016
SAH: Self-assessed of health status
CI: Concentration index
HI: Horizontal inequality index
ICC: Intra-Class Correlation Coefficient
SD: Standard deviation
OR: Odds ratio
